# A Weighty Consideration

**Published:** 1878-06

**Authors:** 


"A WEIGHTY CONSIDERATION.

"Common consumption comes on by slow degrees, and I have never known a case that was not preceded, for months, by an inability of the lungs to measure their full standard. I consider it wholly impossible for a man to have actual consumption, until he has not been able for months to measure the full amount of air. This deficit in the measurement of the lungs *never fails* to exist in any case of clearly defined consumption, and inasmuch as it *always* precedes consumption, its existence for some months in succession ought to be considered a symptom of consumption in its early stages, and a course of treatment should be adopted which would annihilate that deficit at the earliest possible moment.

"To show how certainly this deficit of lung capacity, or lung action, is removed, when it exists not as an effect of a decay of the lungs, but as an effect of imperfect action, I give here a few cases.

"C. W. F., aged 17, an only son of a wealthy family, was placed under my care May 26, 1852. Thin in flesh, pain in side, sore throat, tightness across the breast, short breath, difficult to fetch a long breath, troublesome running and sniffling of the nose, a weak back, with other indications of a weakly constitution. The measurement of his lungs should have been two hundred and twenty-five cubic inches; their actual capacity was two hundred.

| Date. | | Pulse. | Weight. | Breathing. | Lung Measure. |
|---|---|---|---|---|---|
| "May, 1852, | 26, | 72 | 103 | 16 | 200 |
| June | 2, | 72 | 103 | 16 | 206 |
| | 9, | 72 | 103½ | 16 | 216 |
| | 24, | 72 | 107 | 16 | 238 |
| July | 19, | 88 | 104 | 20 | 216 |
| | 23, | 82 | 103 | 18 | 216 |
| August | 7, | 78 | 105 | 15 | 230 |
| | 24, | 76 | 107½ | 16 | 238 |
| Sept. | 29, | 72 | 111½ | 16 | 250 |
| Nov., 1853, | 8, | 72 | 121½ | 16 | 252 |

"The parents of this case, particularly the mother, visited me at different times, expressing the deepest solicitude, and exhibiting an abiding impression that their child, upon whom so many hopes were hung, was certainly going into a decline, especially as he had grown up rapidly, and was a slim, narrow-breasted child.

"The reader will perceive with what admirable promptness the lungs answered to the means used for their development, in the very first fortnight, and with that increase of action a corresponding increase in flesh, so that in four months, and they embracing the hottest of the year, when most persons lose both flesh and strength, he had gained eight and a half pounds, while the capacity of his lungs for receiving air had increased one fifth, that is, fifty cubic inches, and at the end of a year, when he called as a friend, was still gaining in flesh, and strength, and vigor, with no indication, apparent or covert, of any disease whatever.

"What untold treasure would these parents have given, when their child was first brought to me for examination, to have known that the very next year their son would have been one of the most hearty, healthy, manly-looking young men of his age in New York; and yet there can be no doubt that he would have dwindled away, like a flower prematurely withered, had his case been neglected, in the vain hope of his '*growing out of it!*'

"The reader will notice, that on the 13th of July, every symptom became unfavorable; his weight diminished, his breathing was more rapid, and his lung-measurement declined largely. The reason is, that he left the city in June, and spent some weeks at Newport and Saratoga, with his parents, intermitting all remedial means; but, as soon as he returned to New York, and gave diligent attention to what was required of him, his symptoms began at once to abate, and he steadily improved to his recovery. '*The Springs' have proved the grave of many young people with consumptive symptoms*, and older consumptives generally get worse there. The high feeding, or *get what you can* system of diet at watering-places, fashionable hotels, and boarding-houses, their Lilliputian, one-windowed rooms, from one to 'five pair back,' the midnight clatter along interminable passages, the tardy, or no answer, to bell-call, the look-out from your chamber window over some stable, side-alley, or neighbor's back yard; these, with the coldness, and utter want of sympathy at such places, would soon make a well man sick, and will kill instead of cure the consumptive. They want, instead of these, the free, fresh mountain air, the plain substantial food of the country farm house.

